# Diverse Needs of Grandparents Raising Grandchildren: Qualitative Research in Malawi, South Korea, and the United States

**DOI:** 10.1093/geroni/igaa057.2059

**Published:** 2020-12-16

**Authors:** Youjung Lee

**Affiliations:** Binghamton University, State University of New York, Binghamton, New York, United States

## Abstract

Despite custodial grandparents’ significant contributions to their grandchildren’s healthy development, unique needs of older adults often remain unmet with a limited cultural understanding of intergenerational caregiving. Using a phenomenological approach, interviews and focus groups were conducted with 75 custodial grandparents in Malawi (n=29), South Korea (n=23), and the U.S. (n=23). Malawian grandparents presented financial and physical hardships; however, they experienced strong support from community. Korean grandparents reported similar needs as Malawian grandparents while additionally experiencing cultural biases toward grandparent-headed families (maternal grandparenting and adult child’s divorce). The U.S. grandparents disclosed increased needs for social support as well as family trauma with intergenerational impacts. The increase in custodial grandparent population across the world and findings from this comparative transnational research highlight the need for development of a model for culturally responsive practice with grandparent-headed families in a global context. Part of a symposium sponsored by the Grandparents as Caregivers Interest Group.

